# The Associations of Experiencing the COVID-19 Pandemic With Religiosity and Spirituality: A Cross-Sectional Study in Czech Adults

**DOI:** 10.3389/ijph.2022.1604712

**Published:** 2022-06-13

**Authors:** Marie Buchtova, Klara Malinakova, Lukas Novak, Anna Janu, Vit Husek, Jitse P. Van Dijk, Peter Tavel

**Affiliations:** ^1^ Olomouc University Social Health Institute, Palacký University Olomouc, Olomouc, Czechia; ^2^ Graduate School Kosice Institute for Society and Health, University of Pavol Jozef Šafárik, Košice, Slovakia; ^3^ Department of Community and Occupational Medicine, University Medical Center Groningen, University of Groningen, Groningen, Netherlands

**Keywords:** COVID-19 pandemic, behavior, experiences, spirituality, religiosity

## Abstract

**Objectives:** We investigated the associations between religiosity/spirituality and respondents’ changes in their relationships, feelings, thinking, and behaviour during the first wave of the COVID-19 pandemic in the Czech Republic.

**Methods:** A sample of Czech adults (*n* = 1,434; 48.3 ± 16.4 years; 49.65% women) participated in the online survey. We measured spirituality, religiosity, self-reported changes in relationships, disrupted feelings, and changes in behaviour during the pandemic.

**Results:** Spiritual respondents were more likely to report increased physical activity, sex, reading and self-education, with odds ratios (ORs) ranging from 1.26 (95% confidence interval 1.09–1.46) to 1.56 (1.31–1.86). The combination of spirituality and religiosity led to an increase in the range of ORs to 1.57–2.69. Spiritual and religious participants were less likely to feel the decrease of hope by 70%, while mere spirituality significantly reduced the decrease of hope by only 30%. Religiosity itself led to a lower risk of reporting a disrupted day structure with an OR = 0.74 (0.58–0.95).

**Conclusion:** Religiosity and spirituality separately help people during a pandemic in some areas. Especially their combination has a more positive impact on relationships, feelings, and behaviour.

## Introduction

Since its outbreak in December 2019, the new coronavirus SARS-CoV-2 (causing COVID-19) has rapidly spread to become a deadly global pandemic. In addition to the severe threat it poses to human health and to people’s lives, COVID-19 has led to emergency interventions being taken, including restricting people in their homes and closing most businesses [1], as the most frequent way of transmission of the virus is by person-to-person contact [2]. No vaccine was yet available during the studied period.

Evidence suggests that infectious disease epidemics affect not only the physical health of patients but to a large extent also the psychological health and well-being of the non-infected population all around the world [3, 4]. Many people were worried about their family members’ health and safety, financial loss, job loss, and lack of support [5]. An infectious disease is also accompanied by stigmatization [6], which was experienced by citizens who were perceived as the source of the disease [7]. Wang et al. [8] revealed that about one-third of respondents experienced social discrimination caused by the COVID-19 pandemic. A global socioeconomic crisis commenced. Panic and fear of the unknown, resulting in panic buying, hoarding, overwhelming medical centres and health organizations, were reported, as well as the general impact on education, politics, socioeconomics, culture, environment, and climate [4, 9].

Most of the harmful effects of the COVID-19 pandemic can be regarded as risk factors in the development of anxiety, depression, stress, or panic disorder [10–12]. Stress has been previously shown to worsen both physical and mental health, often resulting in increased use of alcohol, tobacco, or other drugs [13]. Social isolation and subjective feelings of loneliness are associated with a higher risk of suicide [14], and unemployment and work restrictions are other factors contributing to the risk of suicide during the COVID-19 period [15].

Furthermore, infectious diseases and a pandemic can represent highly traumatic experiences for some individuals and lead to posttraumatic stress disorder and chronic psychological distress [5]. In some individuals, negative experiences associated with the COVID-19 pandemic may increase the risk of developing psychosocial disorders, such as obsessive-compulsive disorder (OCD) [16], generalized anxiety disorder (GAD) [17], or panic disorder [18], and may increase the occurrence of psychosomatic symptoms [19].

Taken together, during the COVID-19 pandemic we experienced an undeniable negative psychological impact on the general public, and recently, many studies have explored this particular connection (e.g., [10, 20, 21]). However, fewer studies have focused on the protective social and psychological factors that helped to lower the risk of anxiety, depression, and stress (e.g., [22–24]). Evidence indicates that religiosity and spirituality (R/S) can help people to deal with difficult life situations. Religious belief and practice are associated with various health aspects, such as the ability to cope with illness, recovery from hospitalization, or a positive attitude in a challenging life situation [25–27]. Research shows that religious practices may contribute to managing emotions during difficult situations [28], and religiosity, in general, can help a person cope with highly stressful or potentially traumatic events [29, 30]. In the context of the pandemic, R/S can affect health, alleviate suffering and minimize the consequences of social isolation [31]. Positive religious coping, inner religiosity, and trust in a Higher Power can reduce the negative impact of the COVID-19 pandemic, as well as stress [1]. According to Kowalczyk [32], faith is one of the survival strategies that allows one to maintain hope and a sense of security during the current pandemic.

However, religiosity and spirituality have ambiguous meanings and their definitions differ [33]. Religiosity tends to be conceptualized as a social belief and practice related to a higher power, usually associated with a church or organized group [34]. Traditional indicators of religiosity included frequency of church attendance and self-reported levels of religiosity [33]. The concept of religion originally included two dimensions, individual and institutional [35]. However, the individual dimension is now more often labelled as spirituality, that includes the experiences and feelings associated with seeking the sacred, divine, or non-material aspects of life [36]. On the one hand, these two constructs overlap [37], and some authors have suggested conceptualizing a single construct of R/S including institutional and personal dimensions of religion [36]. On the other hand, according to Zwingmann [38], especially in countries with a more secular background, where people often describe themselves as “spiritual but not religious,” it is essential to distinguish between religiosity and spirituality.

Czech Republic is considered one of the most secular societies in the world, and most citizens do not report any religion affiliation [39, 40]. In terms of secularization, the Czech Republic represents a unique environment compared to other European countries due to the significant weakening of the position of religion in history [41]. Rather than religion itself, however, Czechs have a weak relationship with the church as an institution [42], and those who do not affiliate to any organized church should not be seen as atheists, but rather as skeptics who tend to fulfill their religious/spiritual needs outside the organized church [41]. Thus, Czech Republic represents a unique research area, because results in secular countries might differ from those in prevalently religious countries [43]. Therefore, for a more detailed assessment of the effect of R/S on experiences during the pandemic, we decided to explore the associations between R/S and selected variables measuring emotional and behavioural changes, and changes in personal relationships during the first outbreak of the COVID-19 pandemic in the secular environment of the Czech Republic.

## Methods

### Participants and Procedure

We obtained data from an online survey conducted in the Czech Republic during the COVID-19 pandemic in April 2020 to show the current situation in the most stressful period of the first wave of the pandemic. A specialized agency (The Czech National Panel, Prague, Czech Republic) collected data to achieve a balanced sample close to national characteristics regarding gender and age. The inclusion criterion was age 18 years and over. To ensure high data quality, we applied the following exclusion criteria: 1) inconsistencies in control questions relating to participants’ religiosity (feeling the God´s presence despite being non-religious) and 2) a uniform response pattern, i.e., answering a large number of items in the same way. The final sample comprised 1,434 Czech adult respondents (age 18 years and over, mean age = 48.32, SD = 16.44, 49.65% female). From these 1,434 respondents, 1,252 answered all the questions of the online survey.

At the beginning of the survey, respondents were informed in a written form about the purpose of the study and the anonymous and confidential treatment of the data. Specifically, before the survey, they were informed about the content of the survey, their rights and data handling and had to explicitly agree to each of the key points of the informed consent. Electronic informed consent was used because of the nature of the study (an online survey). They then had to click on the appropriate button to indicate their willingness to participate in the survey. The study design was approved by the local Ethics Committee of the Faculty of Theology, Palacký University in Olomouc (No. 2020/06).

### Measures

Religiosity was assessed by the question: “Would you call yourself a believer?” Possible answers were: Yes, I am a member of a church or religious organization; Yes, but I am not a member of a church or religious organization; No; No, I am convinced atheist. Respondents who had reported “No” or “convinced atheist” were classified as non-religious; others were considered religious.

Spirituality was measured using the Daily Spiritual Experience Scale (DSES) [44], which measures the frequency of common experiences of connection with transcendence in daily life. An adapted 15-item version of the scale [39] was used for the present study. Response possibilities for the first 14 items regarded a 6-point scale that ranged from “never” [1] to “many times a day” [6], and for the last item regarded a 4-point scale that ranging from “not close at all” [1] to “as close as possible” [4], leading to total scores from 15 to 88. A higher score of DSES indicates higher spirituality. The reliability (internal consistency) of the DSES was α = 0.96 in our sample. For the purposes of our analysis, the DSES score was treated as continuous. For the assessment of different combinations of religiosity and spirituality with experiencing the COVID-19 pandemic, it was also dichotomized: participants with a score of 51 or higher were considered as spiritual, and the rest as non-spiritual. This cut-off point represents a dichotomization of the total score in the middle (a minimal value is 15, a maximal value 88), and was recently used in the Czech environment [45].

For the last analysis, a composite variable was created based on religiosity and spirituality variables: 1) Non-religious but spiritual, 2) Religious and spiritual, 3) Non-spiritual but religious, 4) Non-spiritual and non-religious.

Experiencing the COVID-19 pandemic was introduced by the following question: “Has anything changed in your life related to the pandemic in the following areas?” followed by 23 items focusing on changes in participants’ lives during the COVID-19 pandemic: a) life with a partner, children, and other people in the household, b) feelings of loneliness, threat, fear and anxiety, helplessness, and hope, day structure, c) frequency of thinking about existential questions and religion, prayer, smoking or chewing tobacco, drinking alcohol, shopping, food consumption, sex, physical activities, reading, self-education, work, telephoning, online communication. For a) and b) the possible answers were: got worse; did not change; got better; the question does not concern me. For c) the possible answers were: I perform this activity less frequently; frequency of this activity did not change; I perform this activity more frequently. The dichotomization was conducted in the following way: for a) and b) the answers “did not change” and “got better” were classified as “not worse,” whereas the answer “got worse” was classified as “worse”; c) The answers “I perform this activity less frequently” and “frequency of this activity did not change” were coded as “not more frequently” and the answer “I perform this activity more frequently” was coded as “more frequently.” The items were chosen based on different life areas and activities that could in general be influenced by the COVID-19 pandemic. Though some of these items might be correlated, we did not expect a mutual relationship between all of them. Therefore, we did not use them as a scale but assessed them as separate variables.

Participants’ socioeconomic status was determined by assigning them to one of the following categories: student, disabled pensioner, employed, self-employed/entrepreneur, homemaker/voluntarily unemployed, unemployed, old-age pensioner, maternity leave.

Age and gender were obtained using the questionnaire.

### Statistical Analyses

First, we used median absolute deviation (MED) to detect low-quality responses. Based on this method, 25 subjects responding inconsistently were deleted. Second, a visual inspection of histograms together with the Mardia test of skewness (standardized multivariate skewness coefficient = 717.78 *p* < 0.001) and kurtosis (standardized multivariate kurtosis coefficient = 7.35 *p* < 0.001) indicated that the normality assumption should be rejected. Thus, non-parametric tests were used in our further analysis. Third, in the logistic regression models, variables assessing a self-reported change of a) relationships and emotionality and b) thinking and behaviour (both related to COVID-19 pandemic) were regressed on religiosity (non-religious/religious). Each model was fitted with a different outcome variable. Numeric variables were standardized to z-scores. All models were adjusted for age, gender, and socioeconomic status, because these variables were reported as important factors mediating other associations (e.g., psychosomatic symptoms) during COVID-19 pandemic. Non-adjusted effects were also reported. Finally, the independent variable (religiosity) was replaced in separate steps by spirituality and a composite variable was created from spirituality and religiosity. In more detail, all models initially fitted using religiosity as an independent variable were fitted again with these new predictors. The R [46] programming software was used for all analyses.

## Results

### Description of the Study Sample

The sociodemographic characteristics of the sample are presented in Table 1. Of the whole sample, 34.5% of respondents were considered religious. The mean spirituality score was 27.6.

**TABLE 1:** Demographic characteristics of the study sample. Czech Republic, 2020.

	N	N (%)
Gender	1,434	
Male		722 (50%)
Female		712 (50%)
Family status	1,434	
In a partnership		492 (34%)
Not in a partnership		942 (66%)
Education	1,434	
Elementary School		116 (8.1%)
Vocational School or Non-Maturity High School		651 (45%)
High School		448 (31%)
Higher Vocational School or University Bachelor		89 (6.2%)
College		130 (9.1%)
Economic status	1,434	
Employed		705 (49%)
Entrepreneur		70 (4.9%)
In household/without work		54 (3.8%)
Pensioner		455 (32%)
Maternity leave		72 (5.0%)
Student		78 (5.4%)
Religiosity	1,434	
Non-religious, convinced atheist		185 (12.9%)
Non-religious		755 (52.6%)
Religious, not a member of church/religious society		371 (25.9%)
Religious, member of church/religious society		123 (8.6%)

### Religiosity

Table 2 shows how the relationships, day structure, emotions, thinking, and behaviour of religious and non-religious participants changed during the COVID-19 pandemic. We found that religious participants had 33% higher odds of deterioration of the feeling of helplessness. On the other hand, they were less likely to report the disrupted structure of the day, with OR = 0.74. Moreover, religiosity was not associated with a lower frequency of health-related behaviours, such as alcohol drinking or smoking, during the COVID-19 pandemic. Religious respondents were 1.74-times more likely to report having sex more frequently during the pandemic than non-religious. Religiosity was associated with more frequent praying and thinking about religion during the pandemic.

**TABLE 2:** Associations of religiosity and changes in relationships, emotions, day structure, thinking and behaviour during the COVID-19 pandemic, crude and adjusted for age, gender, and socioeconomic status (odds ratios and 95% confidence intervals), Czech Republic, 2020.

Changes in relationships, emotions, day structure	Relationship with partner		Relationship with children	Relationship with others in household		Loneliness		Threat		Fear and Anxiety	Helplessness		A decrease of hope	A disrupted structure of a day
Crude effect														
Religiosity	1.12 (0.70–1.78)		1.05 (0.59–1.83)	1.00 (0.55–1.75)		1.31 (0.98–1.74)		1.08 (0.85–1.38)		1.26 (0.98–1.62)	1.46** (1.12–1.88)		0.90 (0.62–1.30)	0.76* (0.60–0.97)
Adjusted														
Religiosity	1.16 (0.71–1.87)		0.98 (0.54–1.74)	1.05 (0.57–1.91)		1.25 (0.92–1.67)		1.01 (0.79–1.29)		1.12 (0.86–1.46)	1.33* (1.02–1.73)		0.84 (0.57–1.22)	0.74* (0.58–0.95)
**Changes in thinking and behaviour**	**Thinking about existential questions**	**Thinking about religion**	**Prayer**	**Smoking or chewing tobacco**	**Alcohol drinking**	**Shopping new things**	**Food consumption**	**Sex**	**Physical activity**	**Reading**	**Self-education**	**Work**	**Calls**	**Other forms of online communication**
Crude effect														
Religiosity	1.06 (0.82–1.36)	11.7*** (6.15–24.5)	13.9*** (7.62–28.1)	0.84 (0.54–1.27)	0.84 (0.55–1.27)	0.61 (0.30–1.14)	0.99 (0.73–1.33)	1.43 (0.93–2.20)	1.32 (0.97–1.78)	1.29 (1.00–1.66)	1.44* (1.05–1.95)	1.41* (1.02–1.95)	1.15 (0.90–1.47)	1.09 (0.86–1.38)
Adjusted														
Religiosity	1.02 (0.79–1.31)	11.2*** (5.85–23.6)	12.9*** (7.01–26.1)	0.94 (0.60–1.44)	0.95 (0.61–1.46)	0.66 (0.32–1.25)	1.02 (0.74–1.40)	1.74* (1.10–2.71)	1.36 (0.99–1.86)	1.20 (0.93–1.56)	1.46 (1.06–2.02)	1.53 (1.09–2.14)	1.04 (0.80–1.34)	1.07 (0.84–1.37)

Notes: *p < 0.05, **p < 0.01, ***p < 0.001.

### Spirituality

In the next step, changes in behaviours, emotions, and relationships were regressed on spirituality. Non-spiritual participants had a 30% higher risk of a decrease of hope. Apart from this, our results indicated that spirituality was not associated with any change in relationships, emotions, or day structure. However, it was associated with increased food consumption, sexual activity, physical activity, reading, self-education, and using various forms of online communication during the COVID-19 pandemic, with odds ratios ranging from 1.22 (1.02–1.47) to 1.56 (1.31–1.86). The odds ratios are reported in Table 3. Lastly, we found that during the COVID-19 pandemic, the odds of thinking about religion and prayer in spiritual individuals were approximately three-times higher than in non-spiritual people.

**TABLE 3:** Associations of spirituality and changes in relationships, emotions, day structure, thinking and behaviour during the COVID-19 pandemic, crude and adjusted for age, gender, and socioeconomic status (odds ratios and 95% confidence intervals), Czech Republic, 2020.

Relationships, emotions, day structure	Relationship with partner		Relationship with children	Relationship with others in the household		Loneliness	Threat		Fear and Anxiety	Helplessness		A decrease in hope		A disrupted structure of a day
Crude effect														
Spirituality	0.90 (0.69–1.14)		1.05 (0.79–1.34)	0.94 (0.69–1.23)		1.14 (0.99–1.31)	1.06 (0.94–1.20)		1.08 (0.95–1.22)	1.10 (0.97–1.25)		0.80* (0.64–0.97)		0.93 (0.82–1.04)
Adjusted														
Spirituality	0.78 (0.56–1.05)		1.01 (0.72–1.40)	0.84 (0.57–1.21)		1.05 (0.88–1.25)	1.05 (0.90–1.21)		0.97 (0.82–1.14)	0.94 (0.80–1.11)		0.70** (0.53–0.91)		0.99 (0.85–1.15)
**Changes in thinking and behaviour**	**Thinking about existential questions**	**Thinking about religion**	**Prayer**	**Smoking or chewing tobacco**	**Alcohol drinking**	**Shopping new things**	**Food consumption**	**Sex**	**Physical activity**	**Reading**	**Self-education**	**Work**	**Calls**	**Other forms of online communication**
Crude effect														
Spirituality	1.03 (0.91–1.15)	2.56*** (2.13–3.09)	3.36*** (2.79–4.09)	0.88 (0.70–1.08)	0.95 (0.77–1.15)	0.97 (0.71–1.28)	1.13 (0.99–1.29)	1.27** (1.05–1.51)	1.27*** (1.11–1.45)	1.26*** (1.12–1.41)	1.45*** (1.28–1.65)	1.18* (1.02–1.36)	1.11 (0.99–1.24)	1.18** (1.06–1.32)
Adjusted														
Spirituality	1.00 (0.86–1.16)	2.00*** (1.60–2.54)	2.89*** (2.29–3.71)	0.89 (0.67–1.16)	0.97 (0.73–1.26)	1.20 (0.80–1.73)	1.22* (1.02–1.47)	1.30* (1.01–1.66)	1.29** (1.08–1.54)	1.26** (1.09–1.46)	1.56*** (1.31–1.86)	1.12 (0.93–1.36)	1.09 (0.93–1.26)	1.25** (1.08–1.44)

Notes: *p < 0.05, **p < 0.01, ***p < 0.001.

### Spirituality and Religiosity: The Combination

**FIGURE 1:**
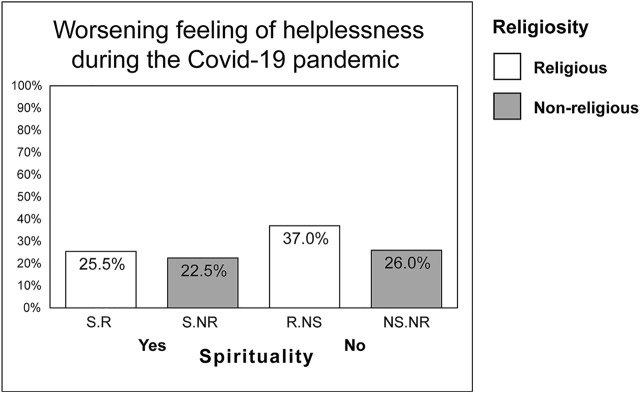
Change in feelings of helplessness in religious and non-religious participants associated with the COVID-19 pandemic (Czech Republic, 2020). (Notes: S.R, spiritual/religious; S.NR, spiritual/non-religious; R.NS, religious/non-spiritual; NS.NR, non-spiritual/non-religious).

Table 4 depicts the associations of different combinations of religiosity and spirituality with changes in relationships, emotions, day structure, thinking, and behaviour during the COVID-19 pandemic. Religious/spiritual respondents were less likely to report a worsening of their feeling of hope (a 70% decrease in the risks). In contrast, religious/non-spiritual participants were 1.48-times more likely to report a deterioration in their feeling of helplessness (see Figure 1 for graphical representation), 1.33-times more likely to report worsening feelings of fear and anxiety and less likely (by 25%) to report the disruption of the day structure.

**TABLE 4:** Associations of different combinations of religiosity and spirituality with changes in relationships, emotions, day structure, thinking and behaviour during the COVID-19 pandemic, crude and adjusted for age, gender, and socioeconomic status (odds ratios and 95% confidence intervals), Czech Republic, 2020.

Changes in relationships, emotions, day structure	Relationship with partner		Relationship with children	Relationship with others in household		Loneliness	Threat		Fear and Anxiety	Helplessness		A decrease of hope		A disrupted structure of a day
Crude effect														
NS.NR	1		1	1		1	1		1	1		1		1
S.R	0.84 (0.32–1.87)		1.23 (0.45–2.80)	0.77 (0.23–1.97)		1.01 (0.57–1.71)	0.67 (0.41–1.06)		0.68 (0.39–1.12)	0.96 (0.58–1.55)		0.36* (0.12–0.82)		0.67 (0.44–1.02)
S.NR	1.29 (0.20–4.60)		3.61* (0.81–11.43)	0.92 (0.05–4.62)		0.75 (0.17–2.28)	0.79 (0.30–1.89)		0.99 (0.35–2.47)	0.80 (0.23–2.27)				0.51 (0.17–1.33)
NS.R	1.15 (0.69–1.87)		1.03 (0.52–1.91)	0.99 (0.52–1.81)		1.37* (1.01–1.86)	1.22 (0.94–1.58)		1.49** (1.13–1.95)	1.62*** (1.22–2.13)		1.07 (0.72–1.57)		0.78 (0.60–1.01)
Adjusted														
NS.NR	1		1	1		1	1		1	1		1		1
S.R	0.81 (0.30–1.84)		1.13 (0.41–2.63)	0.70 (0.20–1.89)		0.96 (0.53–1.66)	0.65 (0.40–1.04)		0.59 (0.34–0.99)	0.87 (0.51–1.42)		0.30* (0.10–0.70)		0.66 (0.42–1.00)
S.NR	1.01 (0.16–3.76)		2.76 (0.59–9.30)	0.72 (0.04–3.80)		0.76 (0.17–2.37)	0.81 (0.30–1.97)		1.03 (0.36–2.63)	0.82 (0.23–2.37)				0.47 (0.15–1.22)
NS.R	1.19 (0.71–1.97)		0.97 (0.49–1.82)	1.10 (0.56–2.06)		1.30 (0.94–1.78)	1.13 (0.87–1.47)		1.33* (1.00–1.76)	1.48** (1.11–1.96)		1.01 (0.67–1.49)		0.75* (0.57–0.98)
**Changes in thinking and behaviour**	**Thinking about existential questions**	**Thinking about religion**	**Prayer**	**Smoking or chewing tobacco**	**Alcohol drinking**	**Shopping new things**	**Food consumption**	**Sex**	**Physical activity**	**Reading**	**Self-education**	**Work**	**Calls**	**Other forms of online communication**
Crude effect														
NS.NR	1	1	1	1	1	1	1	1	1	1	1	1	1	1
S.R	0.74 (0.46–1.15)	27.62 (13.56–61.03)	52.32 (25.85–117.77)	0.97 (0.46–1.84)	1.04 (0.51–1.94)	0.77 (0.23–1.96)	1.41 (0.88–2.20)	2.12* (1.12–3.79)	2.01** (1.27–3.12)	1.71** (1.14–2.54)	2.44 (1.54–3.77)	1.57 (0.91–2.59)	1.06 (0.69–1.60)	1.06 (0.71–1.57)
S.NR	1.21 (0.47–2.83)	23.25 (6.74–72.13)	32.67 (10.10–100.31)	2.23 (0.64–6.04)	3.59** (1.28–8.78)	3.33 (0.76–10.17)	2.11 (0.81–4.95)	3.13* (0.89–8.57)	1.29 (0.37–3.46)	2.04 (0.85–4.60)	3.55** (1.42–8.18)	3.38** (1.29–7.97)	1.48 (0.60–3.37)	1.36 (0.57–3.06)
NS.R	1.20 (0.91–1.58)	6.88 (3.37–15.16)	7.98 (3.84–18.19)	0.79 (0.48–1.26)	0.77 (0.46–1.23)	0.55 (0.24–1.13)	0.85 (0.59–1.20)	1.16 (0.70–1.89)	1.11 (0.77–1.57)	1.16 (0.87–1.54)	1.18 (0.82–1.68)	1.42 (0.98–2.03)	1.19 (0.91–1.57)	1.11 (0.85–1.44)
Adjusted														
NS.NR	1	1	1	1	1	1	1	1	1	1	1	1	1	1
S.R	0.69 (0.43–1.09)	27.15 (13.15–60.67)	51.99 (25.28–118.59)	1.11 (0.52–2.15)	1.13 (0.53–2.19)	0.82 (0.24–2.15)	1.55 (0.93–2.51)	2.69** (1.37–5.01)	2.11** (1.30–3.35)	1.57* (1.04–2.35)	2.38*** (1.48–3.76)	1.67 (0.95–2.81)	0.98 (0.63–1.49)	1.05 (0.69–1.59)
S.NR	1.17 (0.45–2.75)	24.45 (6.95–77.87)	36.96 (11.04–118.48)	1.67 (0.47–4.66)	2.78* (0.94–7.19)	2.76 (0.62–8.79)	1.67 (0.62–4.08)	2.21 (0.61–6.37)	1.05 (0.30–2.87)	2.18 (0.90–4.96)	3.27** (1.28–7.71)	3.09* (1.14–7.56)	1.65 (0.65–3.87)	1.24 (0.50–2.86)
NS.R	1.16 (0.88–1.53)	6.46 (3.14–14.33)	7.13 (3.41–16.35)	0.87 (0.52–1.41)	0.88 (0.52–1.44)	0.60 (0.26–1.25)	0.87 (0.60–1.25)	1.36 (0.80–2.25)	1.15 (0.79–1.64)	1.10 (0.82–1.46)	1.21 (0.83–1.75)	1.55* (1.06–2.25)	1.07 (0.81–1.42)	1.08 (0.82–1.41)

Notes: *p < 0.05, **p < 0.01, ***p < 0.001. S.R, spiritual and religious; S.NR, Spiritual but Non-religious; NS.R, Non-spiritual but Religious. It was not possible to estimate Hope (S.NR) due to the low number of respondents in this category; the regression model did not converge. NS.NR, Non-spiritual and Non-religious.

In spiritual and religious participants, we observed higher chances of more frequent sex, physical activity, reading and self-education, with odds ratios ranging from 1.57 (1.04–2.35) to 2.69 (1.37–5.01). Moreover, spiritual and non-religious respondents were 3.3-times more likely to report more frequent self-education, approximately 2.8-times more likely to report alcohol drinking, and three-times more likely to report more frequent work. The frequency of work was significantly increased (by 55%) among religious and non-spiritual participants.

## Discussion

This study aimed to assess the associations between R/S and respondents’ experiences, behaviour, and relationships during the first outbreak of the COVID-19 pandemic in the Czech Republic in 2020, in the absence of a vaccine. We found that religiosity, spirituality, and their combinations affected experiences, behaviour, and thinking during the pandemic, although the results are heterogeneous. In terms of emotions, R/S had a positive effect on changing feelings of helplessness, hope, a disrupted structure of the day, and fear and anxiety. Regarding behaviour changes, spirituality itself increased the frequency of alcohol drinking, self-education and work. The combination of religiosity and spirituality underlined positive changes in some areas of behaviour and feelings during the pandemic, such as feelings of helplessness, hope, physical activity, sex, reading or self-education.

We found that R/S influenced feelings during the COVID-19 pandemic. Concerning helplessness, fear and anxiety, the absence of spirituality increased these negative emotions. We found that spirituality reduced the odds of decreasing hope. Moreover, in combination with religiosity, the odds were even lower. Our findings seem consistent with Roberto et al. [47], supporting the positive influence of spirituality on hope during the pandemic. Furthermore, our results are similar to those of Lucchetti et al. [31], reporting a positive relationship between R/S and a feeling of hope and a negative relationship between R/S and levels of fear during the current pandemic. Despite the different methodological approach, we came to similar results, which underlines the role of spirituality in promoting positive mental health during stressful situations [48]. Furthermore, religious non-spiritual participants were less likely to report a worsening of the feeling of a disrupted structure of the day. An explanation may be that religious participants are better placed to follow a certain daily and weekly schedule. Religiosity is mostly associated with a system of beliefs, practices and rituals shared in a community [49], and participation in a religious community is usually associated with regularity. Moreover, prayer can play an important role in the structure of the days of religious people [50]. Thus, religious people may have a more internalized structure of time. In connection with the current pandemic, our study suggests that people who already have some religious attitudes can mobilize them when dealing with difficult circumstances [51].

Furthermore, we found that R/S influenced some behaviours during the COVID-19 pandemic. Religious and spiritual participants reported increased odds of physical activity, reading and self-education. In the context of the current pandemic, a positive impact of physical activity and R/S on health has been proven. Spirituality is considered one of the protective factors against the deterioration of mental health outcomes during a pandemic [23, 31, 52]. To the best of our knowledge, this is the first study that found R/S to be associated with higher physical activity during a pandemic. Because recent research prior to the COVID-19 pandemic has not confirmed this particular relationship [53–55], we can assume that it is the current pandemic that is playing a role. A possible explanation may lie in the keeping of religious norms, which, among other things, prompt a person to the care of his or her body. It may also be related to the fact that religion gives meaning to life and thus strengthens life satisfaction and self-esteem [49]. Religious norms offer believers an order on which they can rely.

Moreover, adherence to such an order can also be related to significant changes in other domains, such as reading and self-education. On the other hand, self-education with reading during the pandemic could be associated with greater self-enhancement in religious people [56].

We found that religious and spiritual respondents reported more frequent sex than before the pandemic. Some studies (e.g., [57]) suggest a relationship between spirituality and sexuality during difficult life circumstances. To the best of our knowledge, this is the first study to report an increase in sexual activity in religious and spiritual people during the pandemic. We can assume that this is related to the impossibility of meeting in churches and communities during the COVID-19 pandemic. With the lack of a community, religious people may have had a greater need for close contact, sharing and strengthening relationships in the family, and so they could perceive sex as a form of dealing with this issue.

Concerning spiritual and non-religious participants, we have seen an increase in the odds of drinking alcohol during the pandemic. From the point of view of traumatic situations, this group seems to be more fragile than other R/S subgroups in the Czech environment [58]. Spiritual and non-religious participants may have a higher tendency to look for self-determination and something to rely on, and can therefore fall into alcohol addiction more easily.

In our study, results concerning religiosity were different from those on spirituality. The discrepancy between results related to religiosity and spirituality or different ways of assessment of these constructs has appeared in some previous studies [43, 59, 60]. Moreover, our results suggest that the particular impact of religiosity and spirituality on changes in experience and behaviour during the pandemic was reinforced by the combination of R/S. These findings are in line with some recent research examining differences between R/S subgroups in multiple domains in the Czech environment regarding health-risk behaviour [42, 61] or self-esteem [62]. The results confirm that research on the effect of R/S must be interpreted carefully. Both constructs are multidimensional [63, 64] and so far there is no standard delineations of their definition in the literature [49]. A group of religious participants may include respondents with different levels of spirituality and vice versa [61, 62]; therefore, to achieve relevant results it is essential to consider individual dimensions when measuring R/S.

### Strengths and Limitations

The first strength of this study is that it focuses on the role of R/S during the most critical phase of the first wave of the COVID-19 pandemic. Another strength is a large sample, which is, in terms of age and gender, close to the national sample characteristics. A limitation of our study is its cross-sectional design, so any conclusion on causality cannot be made. Another limitation may be the sampling method, because though the sample was balanced regarding age and gender, some bias is inevitably introduced by the online nature of the questionnaire, which excluded participants without access to the internet. The last limitation can be an information bias, as the survey is based only on the self-report of participants.

### Conclusion

Our findings suggest that religiosity and spirituality have a positive effect during a pandemic. It appears to be a protective factory of negative emotions such as helplessness, fear and anxiety and hopelessness. These results confirm the role of R/S as a potential source of inner strength during difficult life situations. However, R/S does not only affect changes in emotions during a pandemic. The authors point to an association between R/S and increased physical activity and sexual activity during a pandemic, and R/S also contributes to increased reading and self-education.

Although both religiosity and spirituality had an impact on changes in experience and behavior during a pandemic, it is the combination of R/S that reinforced changes in some areas of feelings and behavior during the pandemic. The results of the associations of religiosity and of spirituality with our variables of interest differed among these variables, which means that religiosity and spirituality are not totally overlapping concepts. This idea is also supported by previous studies examining these aspects in secular settings. The results highlight the need to understand R/S as a multifaceted construct and thus eliminate the risk of skewing results by inappropriate research designs.
